# Gold Nanoparticles (AuNPs) Coadministered with a β-Blocker Prevent Liver Fibrosis Caused by Ethanol and Methamphetamine in Rats by Downregulating the Expression of M2 Macrophages

**DOI:** 10.1021/acsomega.4c10118

**Published:** 2025-04-08

**Authors:** Vinícius Barreto Garcia, Luiz H. S. Gasparotto, Aurigena A. de Araujo, Renata F. C. Leitão, Gerly A. C. Brito, Natalia Feitosa Vilar, Emily Lima Oliveira, Paulo M. M. Guedes, Raimundo F. de Araújo Júnior

**Affiliations:** † Inflammation and Cancer Research Laboratory, Department of Morphology, Federal University of Rio Grande do Norte (UFRN), Natal 59078-970, RN, Brazil; ‡ Institute of Chemistry, Federal University of Mato Grosso (UFMT), Cuiaba 78060-900, MT, Brazil; § Department of Pharmacology, Federal University of Rio Grande do Norte (UFRN), Natal 59078-970, RN, Brazil; ∥ Department of Morphology, Postgraduate Program in Morphology, Federal University of Ceará (UFC), Fortaleza 60355-636, CE, Brazil; ⊥ Department of Microbiology and Parasitology, Federal University of Rio Grande do Norte (UFRN), Natal 59078-970, RN, Brazil

## Abstract

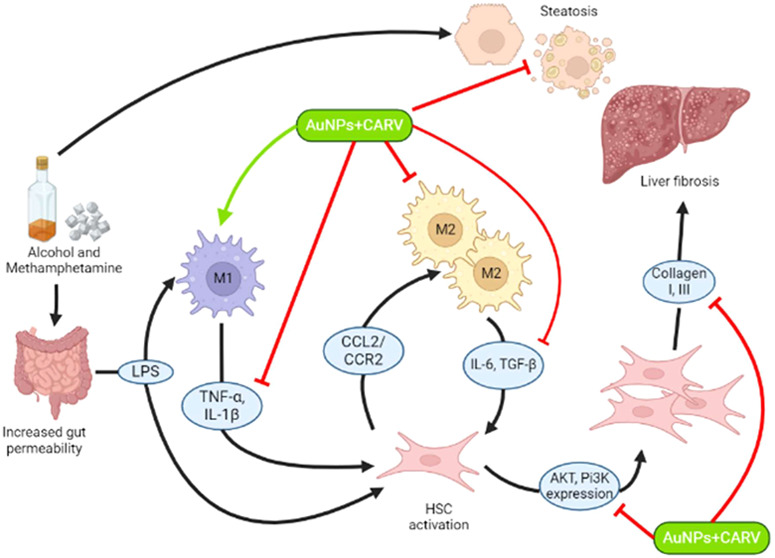

Simultaneous abuse of ethanol and methamphetamine (METH) causes severe liver damage through oxidative stress and inflammation. This study evaluated the antifibrotic effects of gold nanoparticles (AuNPs) coadministered with the β-blocker carvedilol (CARV) against liver damage in rats. Male Wistar rats received 30% ethanol (7 g/kg) daily for 28 days, with METH (10 mg/kg) administered on the 22nd and 28th days. Liver damage was assessed using serum hepatic enzymes, glutathione (GSH) levels, malondialdehyde (MDA) formation, myeloperoxidase (MPO) inhibition, and histopathological analysis, including H&E, Picrosirius Red staining, immunofluorescence, and transmission electron microscopy. Cytokine levels were measured in liver tissue samples. In vitro, RAW 264.7 macrophages were induced to polarize into M1 and M2 phenotypes and cocultured with AuNPs + CARV-treated 3T3 cells, analyzed by rtPCR. AuNPs + CARV effectively protected the liver by modulating interactions between hepatic stellate cells (HSCs) and Kupffer cells, promoting an antifibrotic immune response driven by M1 macrophages. This was indicated by downregulation of profibrotic M2 macrophages and upregulation of M1 macrophages, shown by an increased CD86/CD163 ratio and reduced levels of IL-1β, TNF-α, TGFβ, AKT, and PI3K., pointing an attenuated liver inflammation. These results suggest that AuNPs combined with CARV could potentially serve as a therapy for alcohol and METH-induced liver fibrosis by targeting M2 macrophages.

## 1 Introduction

Alcoholic liver disease (ALD) and methamphetamine-induced liver disease (MILD), which are characterized by progressive inflammation, fibrosis, and hepatocellular damage, present significant obstacles to contemporary healthcare. Ethanol is the most commonly coadministered substance with illicit drugs, significantly contributing to the rise in hospital admissions and fatalities.^1,2^ It is often used alongside psychostimulants like methamphetamine (METH) or cocaine^1,3^ to intensify and extend the effects of these drugs. Liver disorders such as cirrhosis, steatosis, and hepatocellular carcinoma can be brought on by long-term alcoholism and methamphetamine abuse.^4^ Effective therapeutic interventions are urgently needed to mitigate liver injury and promote tissue repair because of the complex interplay between inflammatory processes, fibrogenesis, and immune cell activation.

Recent pharmacological research highlights the therapeutic potential of gold nanoparticles (AuNPs) and carvedilol (CARV) in treating ALD and MILD liver fibrosis and inflammation. In a number of liver injury experimental models, CARV—a nonselective β-blocker with antioxidant and anti-inflammatory properties—has demonstrated promise in reducing liver injury and fibrosis.^5−8^ In a previous study of our group,^5^ CARV suppressed inflammatory markers, including TNFα, IL-1, COX-2, RANKL, RANK, ICAM-1, and IBA-1, while modulating the signaling pathways of Kupffer cells and hepatic stellate cells (HSCs). This resulted in decreased oxidative stress, inflammatory response, and fibrosis in rats with ethanol-induced liver injury.

Furthermore, because of their anti-inflammatory, antioxidant, and antifibrotic qualities, gold nanoparticles have become novel therapeutic agents with potential advantages for the treatment of liver disease. In a previous study conducted by our group, treatment with AuNPs (724.96 μg/kg) was associated with reduced steatosis, hepatic cord degeneration, fibrosis, and necrosis in a rat model exposed to ethanol and METH. Furthermore, a decrease in biochemical markers of oxidative stress, liver damage, and pro-inflammatory cytokines such as TNF-α and IL-1β was observed compared to the group treated with ethanol and METH alone. Moreover, a decline in the expression of FGF, SOD-1, and GPx-1 was noticed. The study came to the conclusion that AuNPs affected the pro-inflammatory cytokine profile, oxidative stress, and fibrosis of Kupffer cells and hepatic stellate cells by modulating the signaling pathways AKT/PI3K and MAPK.

Central to the pathogenesis of ALD and MILD are the dynamic interactions between immune cells, particularly M1 and M2 macrophages, which play pivotal roles in orchestrating the inflammatory response and tissue repair processes in the liver.^9^ Macrophage polarization is essential for numerous pathological processes, including inflammation, tissue repair, and tumor growth,^10−12^ leading to the onset of various liver conditions, such as fatty liver disease, hepatitis, fibrosis, and hepatocellular carcinoma.^13−15^ The main effect of M1 macrophages is known to be proinflammatory. They can activate adaptive immune responses and generate proinflammatory and stress mediators, as well as cytokines such as TNFα, IL-1, IL-12, IL-18, interferon γ, nitric oxide, and reactive oxygen species. To limit excessive tissue-damaging inflammatory responses, macrophages transform into a tissue-restorative, anti-inflammatory phenotype once the infection or injury is under control. Usually known as alternatively activated macrophages (M2), these cells support both tissue repair and the reduction of inflammation.^16^

In this regard, the role of various macrophage phenotypes in liver diseases remains controversial, particularly due to the difficulty of modulating their behavior in inflammatory environments. Targeting these cells using a combination of spherical gold nanoparticles and a β-blocker appears to be a promising therapeutic approach.^17^ This study seeks to investigate the ability of gold nanoparticles, when combined with carvedilol, to reduce inflammation and prevent fibrosis in a rat model of liver injury induced by alcohol and methamphetamine. Without functionalization, bare AuNPs do not have specific targeting capabilities and might not effectively accumulate in fibrotic liver tissue. This nonspecific distribution can reduce their therapeutic efficacy and increase the risk of off-target effects. Combining bare AuNPs with other therapeutic strategies, such as coadministration with antifibrotic drugs, might enhance their efficacy. This approach could leverage the inherent properties of AuNPs while compensating for their lack of targeting. By comprehensively understanding the molecular mechanisms underlying the actions of AuNPs and carvedilol and their interactions with macrophages 1 and 2 in the context of ALD and MILD, this study seeks to provide insights into novel therapeutic strategies for liver disease management.

## 2 Results

### 2.1 In Vitro Assays

#### 2.1.1 Viability Assay

**Figure 1:**
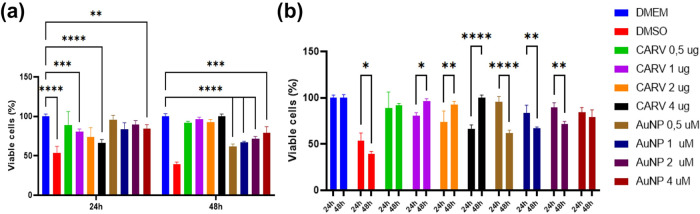
Viability assay of CARV and AuNPs on 3T3 cells. The toxic effects of CARV and AuNPs were assessed using the Trypan Blue assay, with the graphical representation showing the percentage of viable cells at both 24 and 48 h. Comparisons were made either between treatment groups and the DMEM control group (a) or between different time points within the same treatment (b). All data are reported as mean ± standard deviation (SD) from four independent experiments, each conducted with at least two replicates. Statistically significant differences are indicated by multiplicity-adjusted p-values: *p < 0.05, **p < 0.005, ***p < 0.001, and ****p < 0.0001, analyzed using two-way ANOVA followed by post hoc Dunnett’s (a) or Sidak’s (b) correction.

Exclusion of trypan blue viability assay was done in order to determine the lowest doses of CARV and AuNPs that effectively reduce cell viability of 3t3 cells and, consequently, impair profibrotic immune responses. As it is shown in Figure 1, CARV and AuNPs appear to have some complementary effect, with CARV showing its effects in the first 24 h, and AuNPs from 48 h. CARV reduced 3t3 cells viability by about 20 and 40% in the first 24 h at the doses of 1 and 4 μg/mL, respectively (Figure 1a). Although AuNPs only proved to be effective in its highest dose in 24 h (**p < 0.005), in 48 h, every dose tested significantly reduced cell viability in about 35% (****p < 0.0001) (Figure 1a).

#### 2.1.2 M1/M2 Macrophage Polarization

**Figure 2:**
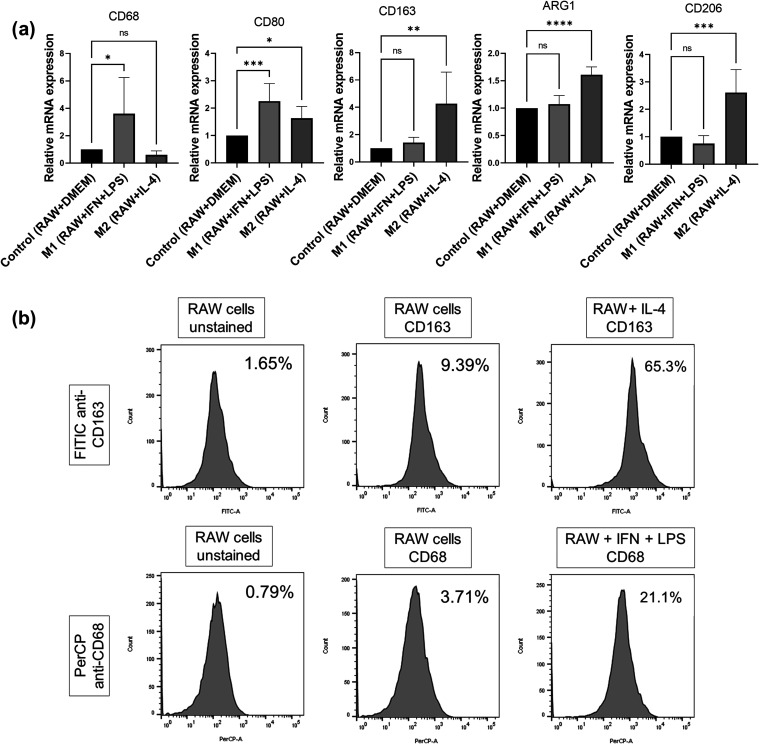
M1 and M2 macrophage characterization by qPCR (a) and Flow Cytometry (b). The M1 phenotype is characterized by CD68 and CD80 gene expressions, while CD163, ARG1, and CD206 gene expressions are present in the M2 macrophage phenotype. All results are expressed as mean ± standard deviation (SD) from two independent experiments, each performed with a minimum of three replicates. Statistically significant differences are indicated by multiplicity-adjusted *p*-values: *p < 0.05, **p < 0.005, ***p < 0.001, and ****p < 0.0001; (ns) denotes no significant difference. Data were analyzed using one-way ANOVA followed by post hoc Dunnett’s correction.

The polarization of RAW 264.7 macrophages into M1 and M2 phenotypes was verified through the expression of CD68, CD80, CD163, ARG1, and CD206 genes in the qPCR assay, and by CD68^+^ and CD163^+^ phenotypes shown in the flow cytometry assay. As the qPCR analysis shows, 48 h treatments with IFNγ/LPS and IL-4 successfully induced M1 and M2 macrophage polarization, respectively (Figure 2a). CD68 gene expression was increased 4-fold in RAW 264.7 cells exposed to IFNγ/LPS compared to RAW 264.7 cells cultured in DMEM alone or treated with IL-4 (*p < 0.05, Figure 2a). Likewise, IFNγ/LPS treatment doubled the CD80 expression (***p < 0.001, Figure 2a). Regarding M2 polarization, IL-4 markedly upregulated the mRNA expression of CD163, ARG1, and CD206 in RAW 264.7 cells (Figure 2a). In the flow cytometry assay, IL-4 raised CD163+ cells by 65.3%, and IFN+LPS increased positive CD68+ cells by 21.1% (Figure 2b).

#### 2.1.3 CARV and AuNPs Uptake Assay

**Figure 3:**
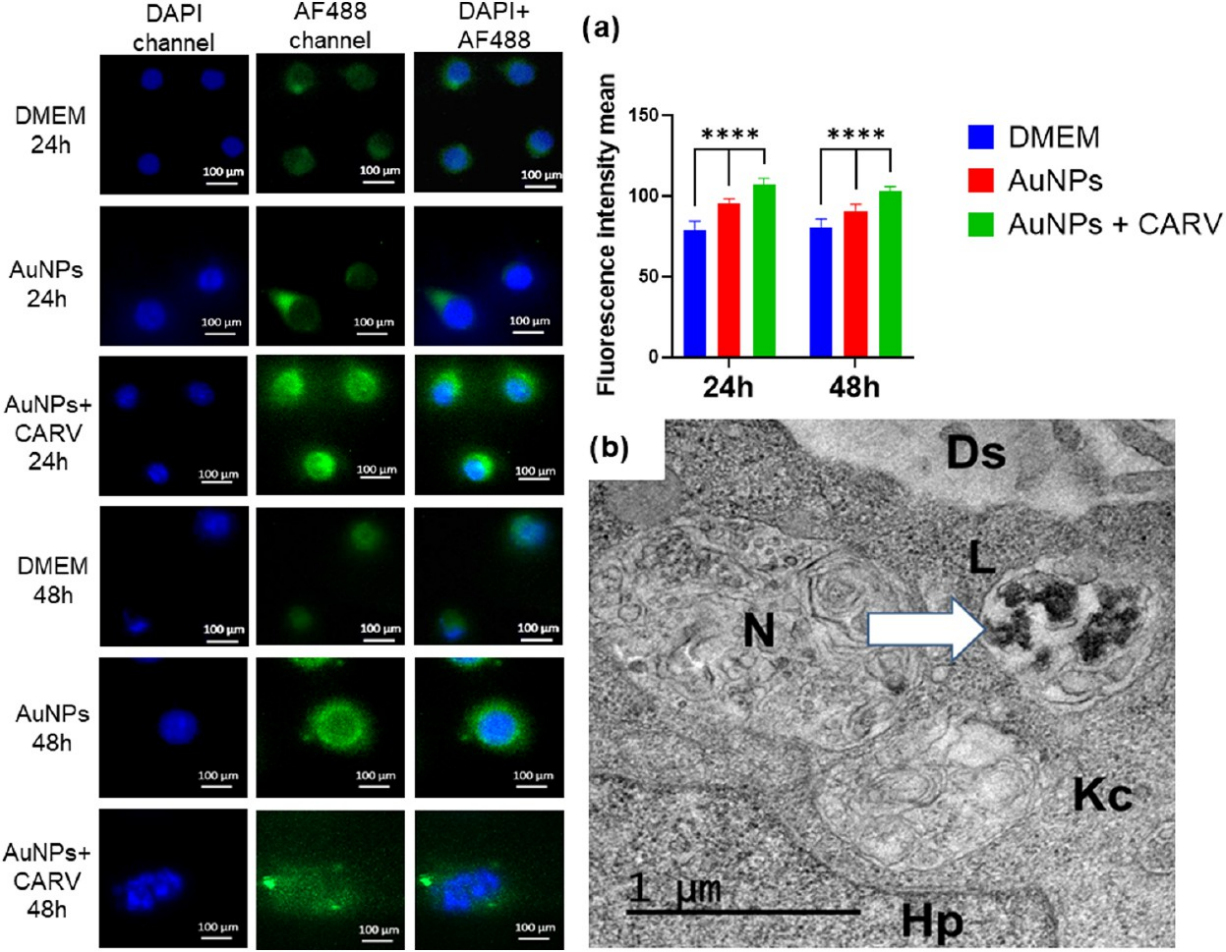
Cellular uptake of AuNPs and CARV assessed by immunofluorescence and TEM. RAW 264.7 cells were incubated with AuNPs (1 μM/mL) and CARV (1 μg/mL) for 24 and 48 h. Fluorescence microscopy images were captured using DAPI and Alexa Fluor 488 (AF488) channels to detect nuclear staining (DAPI, blue) and AuNPs (green), respectively. The mean fluorescence intensity for each treatment was used to compare AuNPs- and CARV-exposed cells with the DMEM control group (a). In the TEM image (b), the white arrow indicates AuNPs + CARV within the phagosome of a Kupffer cell (Kc) in liver tissue. Data are presented as mean ± SD from six independent experiments, each performed with at least two replicates. ****p < 0.0001, denoting significant differences as indicated; statistical analysis was performed using two-way ANOVA with Tukey’s (a) and Sidak’s (b) post hoc tests.

The uptake rate of AuNPs and AuNPs+CARV by RAW 264.7 macrophage cells was evaluated through fluorescence intensity quantification and TEM analysis. As depicted in Figure 3, the findings demonstrate that both AuNPs and AuNPs + CARV were successfully internalized by macrophage cells following 24 and 48 h treatments. However, AuNPs + CARV were significantly better internalized (Figure 3a,b) than AuNPs alone. The comparison between the 24 and 48 h periods is provided as Supporting Information (Figure S1).

#### 2.1.4 NFκB, TGFβ, and IL-10 Gene Expression by 3t3 Cells

**Figure 4:**
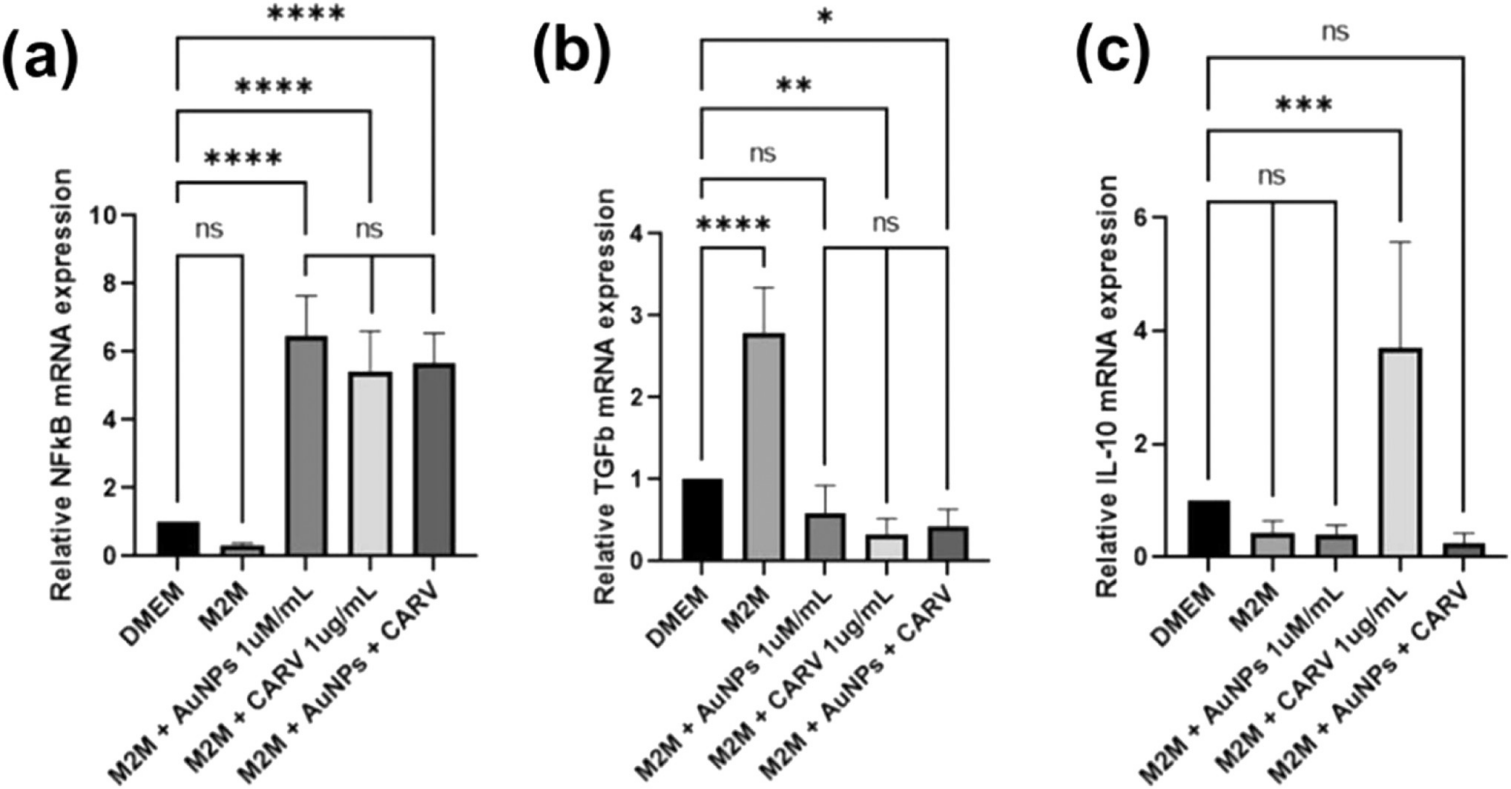
Relative mRNA expression of NFkB (a), TGFb (b), and IL-10 (c) by 3t3 cells cultured in FBS-free DMEM and M2-polarized TAMs supernatant-enriched medium (M2M) treated with AuNPs (1 μM/mL), CARV (1 μg/mL), and a combination of AuNPs + CARV. Data are presented as the mean ± SD from two independent experiments, each with at least three replicates. Statistically significant differences are indicated as *p < 0.05, **p < 0.005, and ****p < 0.0001, while (ns) denotes no significant difference. Statistical analysis was performed using one-way ANOVA followed by Tukey’s post hoc correction.

In order to investigate if CARV, AuNPs, and their association may interfere in the activation mechanisms behind fibroblastic cells in a M2-managed microenvironment, 3T3 cells were cultured and treated in FBS-free DMEM supplemented with the enriched supernatant of M2-polarized TAMs (M2M), as previously described. As the results show, both the AuNPs and CARV increased NFκB expression up to 6x in comparison to nontreated 3t3 cells (****p < 0.0001, Figure 4a), antagonizing M2M effects on these cells. No significant difference was found in NFκB or TGFβ gene expression among the treated groups (Figure 4). Curiously, only CARV seems to stimulate IL-10 expression by 3t3 (***p < 0.001, Figure 4c).

### 2.2 In Vivo Assays

#### 2.2.1 Biochemical Assays For Liver Damage

**Figure 5:**
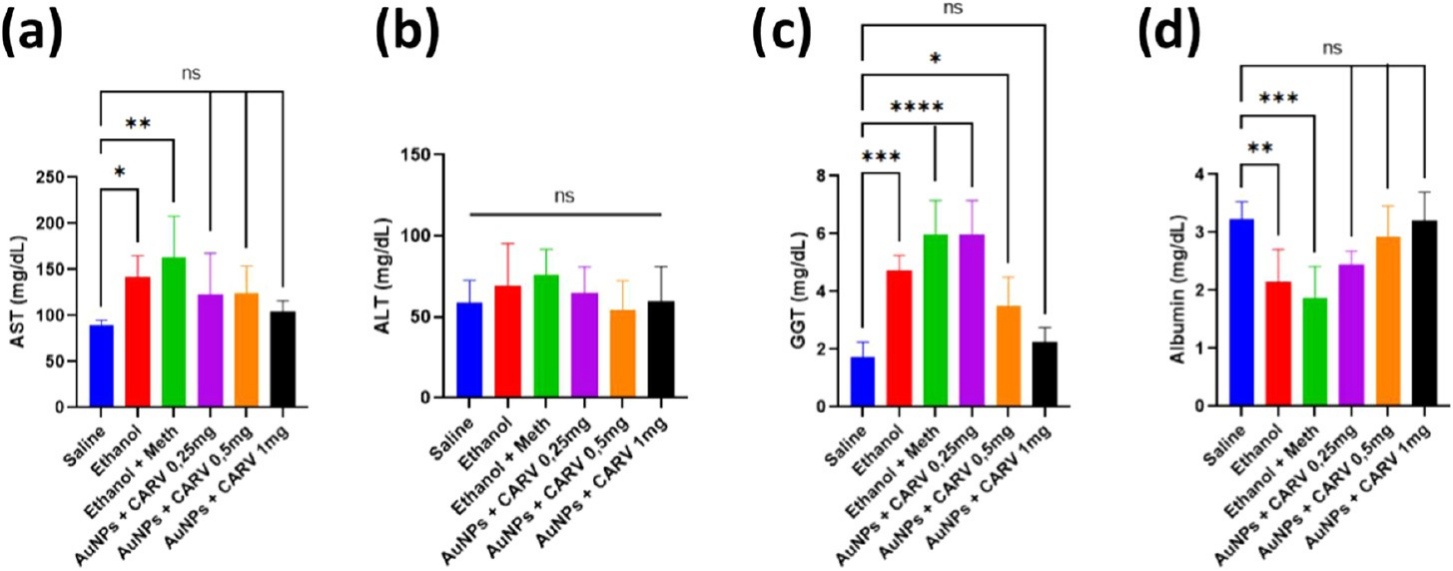
Serum levels of AST (a), ALT (b), GGT (c), and Albumin (d) in Wistar rats submitted to alcoholic liver injury. All data are expressed as mean ± SD. Each group consisted of 6 animals (*n* = 6). Statistically significant differences are indicated as *p < 0.05, **p < 0.005, ***p < 0.001, and ****p < 0.0001, while (ns) denotes no significant difference. Statistical analysis was performed using one-way ANOVA followed by Dunnett’s post hoc correction.

To assess liver injury caused by alcohol and meth, blood samples were collected through cardiac puncture to measure the levels of aspartate aminotransferase (AST), alanine aminotransferase (ALT), γ-glutamyl transferase (GGT), and albumin. AST, GGT, and Albumin levels were compatible with what is expected for alcohol-induced damage (Figure 5a,c,d). Curiously, none of the groups showed any significant difference in ALT levels in comparison to the saline group (Figure 5b). In contrast, all treatments involving AuNPs + CARV, particularly at 1 mg/kg, demonstrated a protective effect against ethanol-induced disease (Figure 5c,d), with a distinct dose-dependent trend observed in GGT levels (Figure 5c).

#### 2.2.2 GSH, MDA, and MPO Assays

**Figure 6:**
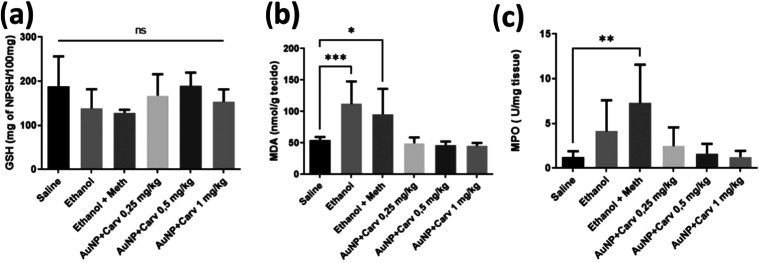
GSH (a), MDA (b), and MPO (c) levels in liver samples of Wistar rats. All data are presented as mean ± SD. There were 6 animals per group (*n* = 6). Statistically significant differences are marked as *p < 0.05, **p < 0.005, and ***p < 0.001, while (ns) indicates no significant difference. Statistical analysis was conducted using one-way ANOVA followed by Dunnett’s post hoc correction.

In order to investigate the impact of the AuNP+CARV treatment on oxidative stress, GSH, MDA, and MPO levels in liver samples were analyzed. Although none of the groups showed significant difference when it comes to GSH levels (Figure 6a), the treatment with AuNPs+CARV, in all doses, was effective in keeping the MDA and MPO levels normal (Figure 6b,c).

#### 2.2.3 Cytokine Analysis

**Figure 7:**
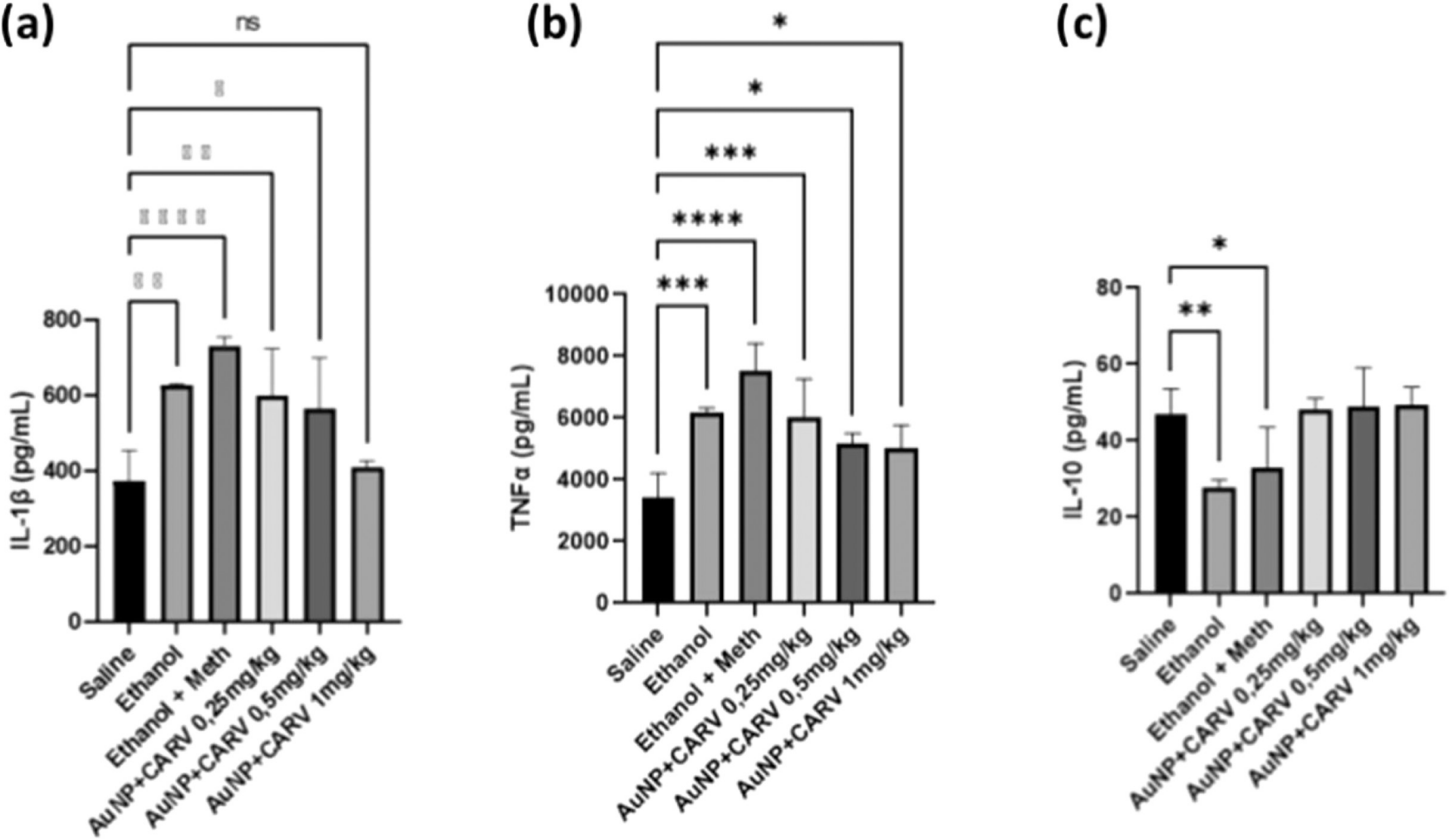
Levels of IL-1β (a), TNFα (b), and IL-10 (c) in tissue samples from Wistar rats with alcohol-induced liver injury. Data are expressed as mean ± SD, with 6 animals per group (*n* = 6). Statistically significant differences are denoted as *p < 0.05, **p < 0.005, ***p < 0.001, and ****p < 0.0001, while (ns) indicates no significant difference. Statistical analysis was performed using one-way ANOVA followed by Dunnett’s post hoc correction.

Cytokine analysis of the tissue shows the immune patterns behind the alcoholic liver damage. As expected, alcohol and alcohol + meth groups showed increased levels of both IL-1β and TNFα proinflammatory cytokines (Figure 7a,b) in comparison to the saline control group (****p < 0.0001). Although only the highest dose of CARV (1 mg/kg) was able to reduce IL-1β down to normal levels (Figure 7a), all AuNPs + CARV-treated groups lowered the levels of both proinflammatory cytokines in comparison to alcoholic groups. In agreement with these results, alcoholic groups showed low levels of IL-10 (**p < 0.005, Figure 7c), an anti-inflammatory cytokine, while CARV-treated groups managed to keep this cytokine level normal (Figure 7c).

#### 2.2.4 Histopathological Analysis

**Figure 8:**
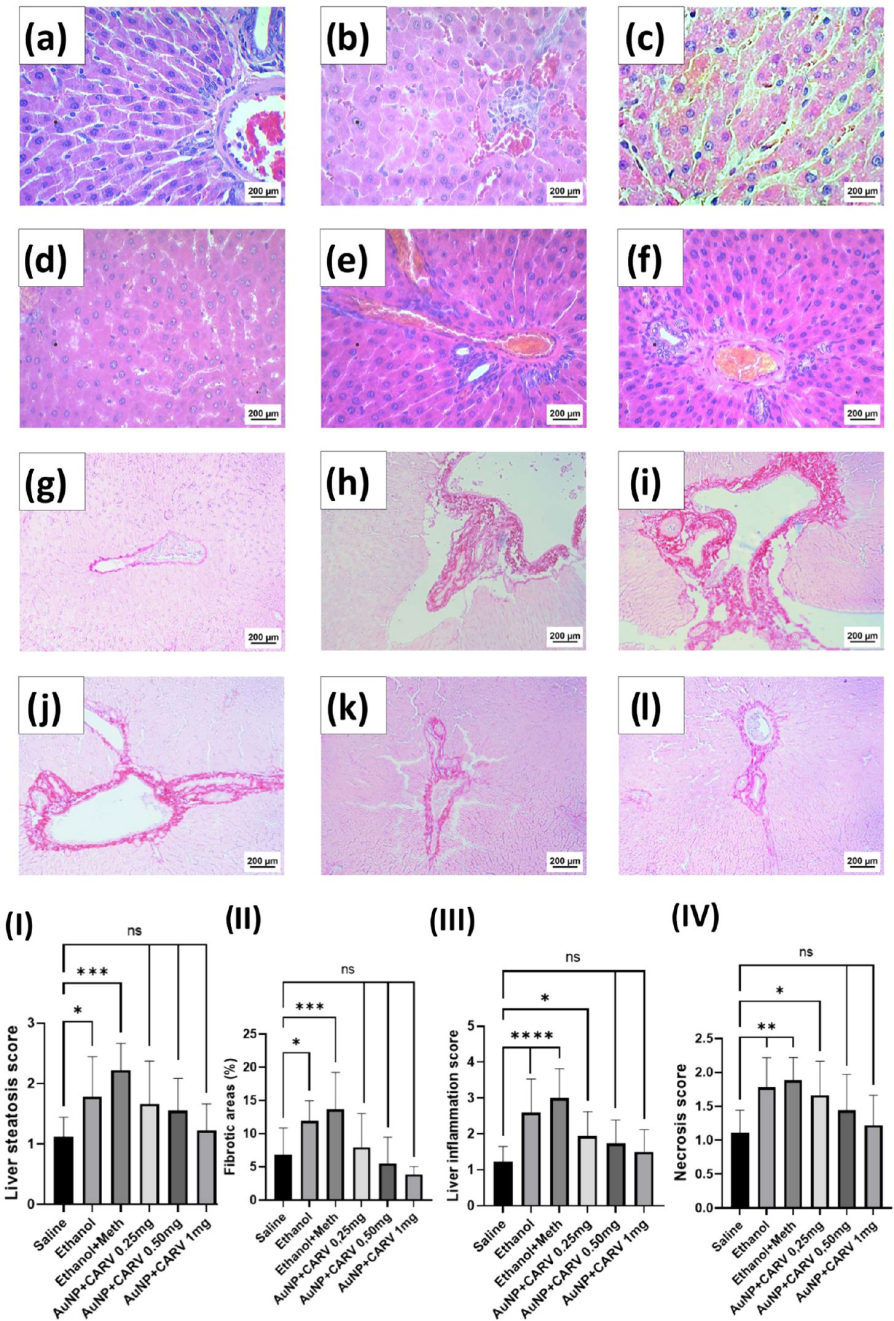
Histopathological evaluation of Wistar rats’ livers submitted to alcoholic and methamphetamine injury. Steatosis (I), fibrosis (II), inflammation (III), and necrosis (IV) were taken as steatohepatitis parameters for the analysis. Hematoxylin & Eosin (a–f) and Picrosirius (g–l) representative images are shown from each of the groups: saline (a, g), ethanol (b, h), ethanol + meth (c, i), AuNP+CARV 0,25 mg (d, j), AuNP+CARV 0,5 mg (e, k), and AuNP+CARV 1 mg (f, l). All data are reported as mean ± SD, with six independent measurements per group (*n* = 6). Statistically significant differences are indicated as *p < 0.05, **p < 0.005, ***p < 0.001, and ****p < 0.0001, while (ns) denotes no significant difference. Statistical analysis was conducted using one-way ANOVA with Dunnett’s post hoc correction.

Histopathological analysis of alcohol and alcohol+meth groups showed typical alterations of steatohepatitis, like fatty degeneration (steatosis) combined with chronic inflammatory infiltrate and necrosis (Figure 8b,c). Picrosirius staining of those groups also showed a substantial increase in fibrotic areas around portal veins (Figure 8h(I,II), ***p < 0.001), whereas AuNP + CARV-treated groups showed normal liver histology when compared to the saline control group.

#### 2.2.5 Immunofluorescence Microscopy

**Figure 9:**
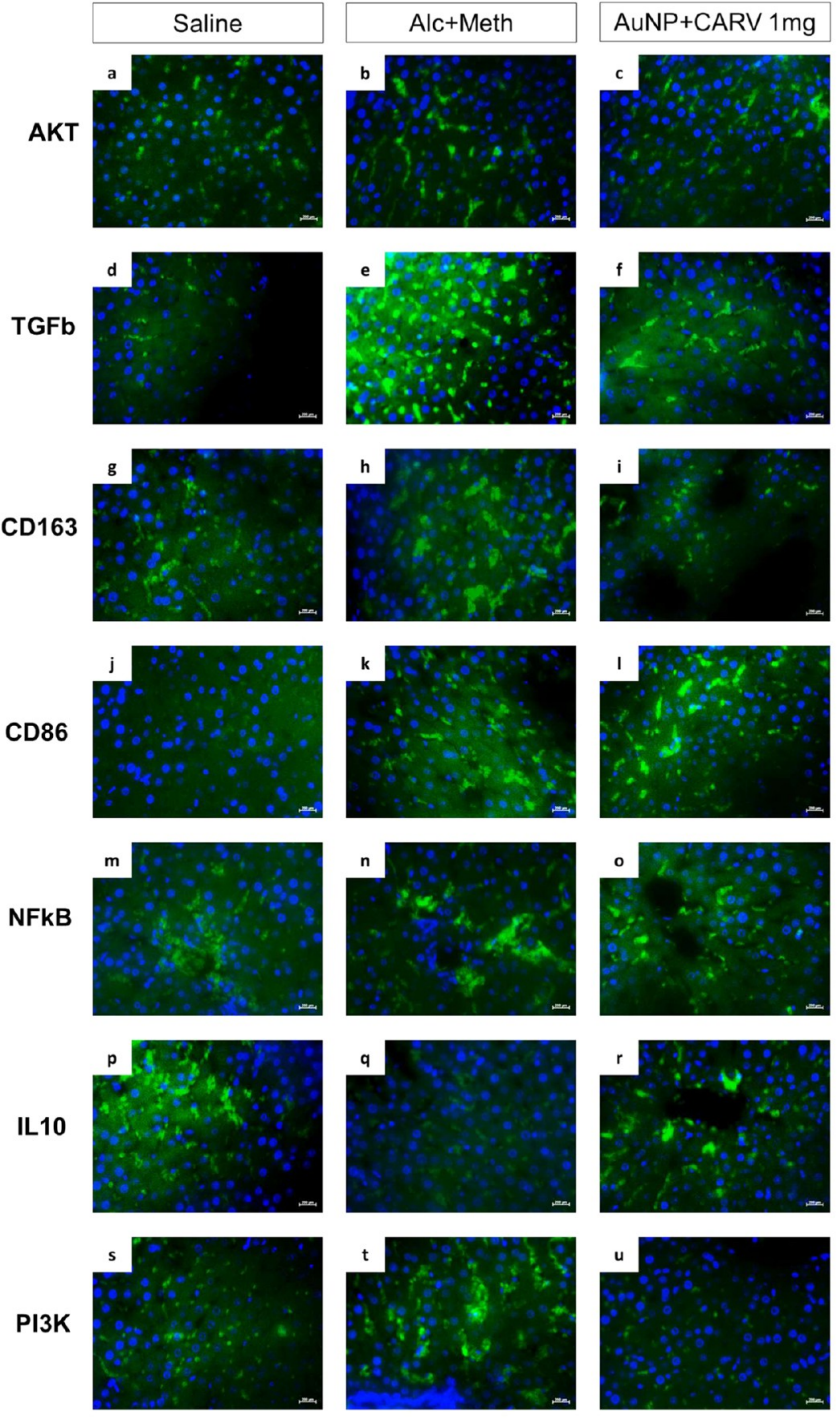
Liver expression of AKT (a–c), TGFb (d–f), CD163 (g–i), CD86 (j–l), NFkB (m–o), IL-10 (p–r), and Pi3K (s–u) in Wistar rats submitted to alcoholic liver injury.

**Figure 10:**
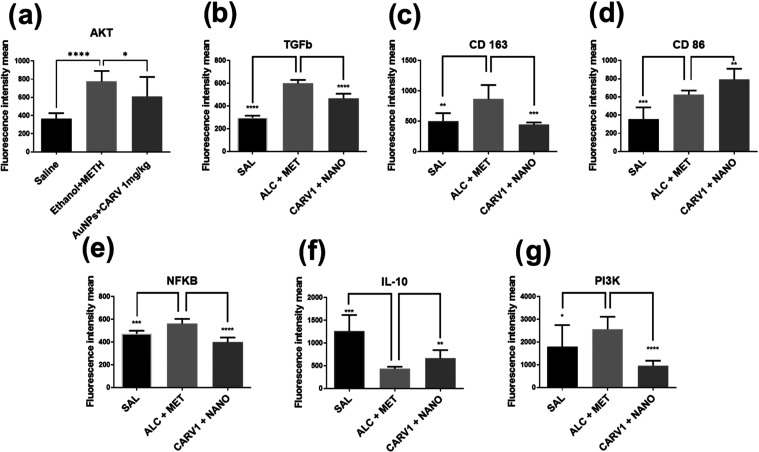
Liver expression of AKT (a), TGFb (b), CD163 (c), CD86 (d), NFkB (e), IL-10 (f), and Pi3K (g). All data are presented as mean ± SD. Fluorescence intensity data was generated by Zeiss Zen Lite software for each picture taken (10 images per subject, 5 animals per group, 400× magnification). Statistically significant differences are indicated as follows: *p < 0.05, **p < 0.005, ***p < 0.001, and ****p < 0.0001; (ns) denotes no significant difference. Statistical analysis was performed using one-way ANOVA with Dunnett’s post hoc correction for multiple comparisons.

**Figure 11:**
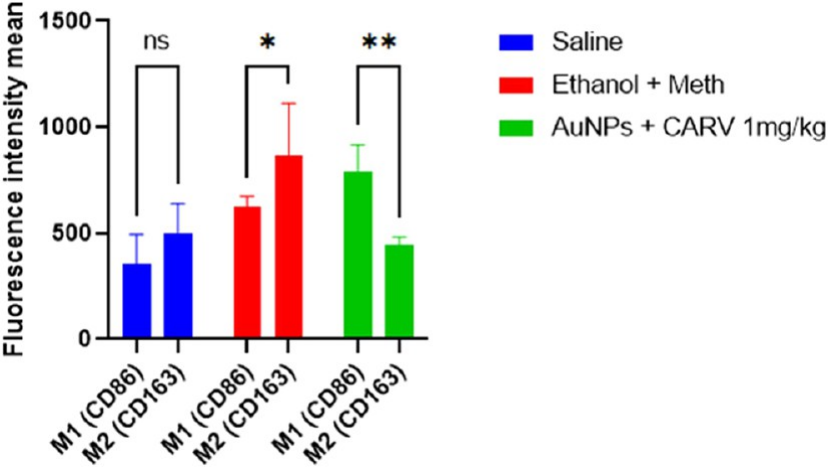
Comparison between M1 (CD86) and M2 (CD163) immunofluorescence labeling in Wistar rats submitted to alcoholic liver injury. All data are presented as mean ± SD. Fluorescence intensity measurements were obtained using Zeiss Zen Lite software, with 10 images captured per subject, 5 animals per group, at 400× magnification. Statistically significant differences are denoted as *p < 0.05 and **p < 0.005; (ns) indicates no significant difference. Data were analyzed using two-way ANOVA with Sidak’s post hoc correction.

Immunofluorescence was performed to identify in the liver tissue of saline, ethanol+meth, and AuNP + CARV 1 mg/kg groups, key proteins related to M1 and M2-mediated immune responses, as well as cell survival and proliferation. Results showed that the AuNP+CARV treatment increased the expression of CD86 (**p < 0.005, Figures 9l and 10d), IL-10 (**p < 0.005, Figures 9r and 10f) and decreased the expression of AKT (*p < 0.05, Figures 9a and 10a), TGFβ (***p < 0.001, Figures 9b and 10b), CD163 (***p < 0.001, Figures 9i and 10c), NFκB (****p < 0.0001, Figures 9o and 10e), and Pi3k (****p < 0.0001, Figures 9u and 10g). As the comparison between CD86 and CD163 fluorescence intensities showed (Figure 11), the expression of CD86 was higher than CD163 one in the treated groups (**p < 0.005), whereas alcohol + methamphetamine induced higher expression of CD163 (*p < 0.05).

## 3 Discussion

CARV works by inhibiting sympathetic neural activation through the blockade of β1-, β2-, and α1-adrenoreceptors. Its antioxidant, anti-inflammatory, and antifibrotic effects on cardiovascular diseases are well-known, both in human and animal studies.^18,19^ In ALD induced in rats, CARV effectively reduced oxidative stress, inflammatory response, and fibrosis by inhibiting the signaling of Kupffer cells and hepatic stellate cells (HSCs) through the suppression of inflammatory cytokines,^20^ such as TGFβ.

In this study, the combination of CARV and AuNPs demonstrated significant protection of liver parenchyma against injury induced by alcohol and methamphetamine. This protective effect was achieved by regulating the interaction between hepatic stellate cells (HSCs) and Kupffer cells (KCs), which promoted an antifibrotic immune response and a controlled proinflammatory process, primarily mediated by M1 macrophages.

Chronic alcohol consumption is directly associated with steatosis (fatty degeneration in hepatocytes), steatohepatitis (when steatosis is associated with inflammation), fibrosis, and hepatocellular carcinoma (HCC).^21^ Steatosis is the first sign of hepatic injury, and it is caused both by acetaldehyde and unbalancing expression of SREBP and PPAR-α.^21^ The main histomorphology characteristic of steatosis is the ballooned hepatocytes filled by lipids vesicles. This condition is reversible if exposure to the harmful agent, in this case ethanol, is halted. However, with chronic injury, it can progress to steatohepatitis and fibrosis, characterized by endoplasmic reticulum stress, mitochondrial damage, hepatocyte apoptosis, inflammatory infiltrates (particularly in portal areas), the release of proinflammatory cytokines such as TNF-α, IL-6, and IL-1β, and the activation of HSCs into myofibroblasts that produce type I collagen.^21^ Most of these events are also present in meth-induced liver injury, which is also known by the cell cycle arrest and overexpression of Cidea, cleaved caspase 3, and PARP1, leading to apoptosis of hepatocytes.^22^

As histopathological analysis of our study shows, the AuNP + CARV combination had both anti-inflammatory and antifibrotic effects, preserving the overall morphology of hepatocytes and liver sinusoids, whereas untreated alcoholic groups exhibited signs of steatohepatitis and fibrosis. Similar results were seen in our previous studies when CARV^5^ and AuNPs^1^ were tested alone in similar animal models. The current findings align with elevated levels of ALT, AST, and GGT in the alcoholic groups, which are strong indicators of liver injury.^23^ AuNPs + CARV treatment prior to alcohol/meth administration proved to be protective against these substances. Along with liver enzyme levels, oxidative stress and cytokine assays revealed the impact that both alcohol and METH had on the animals’ livers, but none of the AuNPs/CARV-treated groups showed high levels of MDA and MPO, indicating that the treatment prevented lipid peroxidation, mitochondrial degeneration, and acute inflammatory infiltrate, all important indicators of steatohepatitis.^21^

In alcoholic liver disease, ethanol increases gut permeability, leading to a scenario of endotoxemia driven by lipopolysaccharides (LPS) from commensal microbiota.^24,25^ LPS activates Kupffer Cells (KCs) through TLR4^26,27^ and NF-κB expression, resulting in the production of proinflammatory cytokines, including IL-1β and TNF-α.^28^ Our examination of cytokine levels in liver biopsies revealed that the alcoholic groups had significantly higher concentrations of TNF-α and IL-1β, coupled with a notable decrease in IL-10. Once the animals were treated with AuNPs+CARV (1 mg/kg), IL-1β levels were decreased down to normal levels (Figure 7a), and IL-10 was maintained at normal levels (Figure 7c). Although TNF-α levels were still high in comparison to the saline group, AuNPs + CARV-treated groups showed lower levels of this cytokine in comparison to alcoholic groups. TNF-α and IL-1β cytokines are usually associated with M1-mediated immune responses^9^ and IL-10 cytokine with M2 macrophages.^29^ Nonetheless, as the immunofluorescence analysis showed, liver inflammation in our study was predominantly driven by M2 macrophages, which effectively internalized AuNPs+Carv as both in vitro and TEM in vivo uptake assays show. This is attested by the increased expression of CD163 over CD86 and high expression of TGFβ in the ethanol+METH group. This fact is very intriguing once M2 activity in ALD is associated with better outcomes.^9^

Reducing pro-inflammatory cytokines IL-1β and TNFα is critical for mitigating acute inflammation and slowing liver fibrosis progression. These cytokines drive the inflammatory cascade, recruiting immune cells like neutrophils and macrophages, which worsen tissue injury and fibrosis. Elevated IL-1β and TNFα levels are associated with sustained inflammation, hepatocyte apoptosis, and hepatic stellate cell activation, leading to excessive extracellular matrix production.^30^ Lowering their levels reduces inflammation, protects hepatocytes, and curbs fibrogenic pathways, potentially halting or reversing fibrosis.

Maintaining IL-10 levels is crucial for immunoregulation and tissue repair in liver disease. IL-10, an anti-inflammatory cytokine, prevents excessive tissue damage by inhibiting pro-inflammatory cytokine synthesis, reducing macrophage activation, and promoting a shift toward the reparative M2 macrophage phenotype. Clinical studies highlight IL-10 gene therapy’s ability to reduce hepatic fibrosis, decrease HSC activation, and improve liver function by downregulating fibrogenic gene expression.^31^ Additionally, IL-10 treatment in chronic hepatitis C patients has improved liver histology and reduced fibrosis, demonstrating its therapeutic potential.^32^ The findings of the present study underscore the importance of balancing pro-inflammatory cytokine reduction, such as IL-1β and TNFα, with IL-10 enhancement as a dual strategy to mitigate inflammation, slow fibrosis progression, and promote tissue repair, offering significant promise for managing chronic liver diseases.

Traditionally, M2 macrophages are known for their anti-inflammatory properties and role in tissue repair. However, their contribution to inflammation and fibrosis in alcoholic liver disease (ALD) has become increasingly evident. In ALD, M2 macrophages can promote and sustain inflammatory processes by secreting pro-inflammatory mediators such as interleukin-1 β (IL-1β), interleukin-6 (IL-6), and tumor necrosis factor-α (TNF-α), which intensify liver inflammation and exacerbate tissue injury. A study by Seidman et al. has demonstrated that M2-like macrophages in ALD are associated with increased levels of IL-6-α and TNF, contributing to the inflammatory milieu in the liver.^33^ M2 macrophages can also contribute to fibrosis progression in ALD by stimulating hepatic stellate cell (HSC) activation and collagen deposition. Several studies have shown that M2 macrophages produce profibrotic mediators, such as platelet-derived growth factor (PDGF) and transforming growth factor-β (TGF-β), which promote HSC activation and collagen synthesis.^34−36^ Stutchfield et al. demonstrated that M2 macrophages in ALD promote liver fibrosis by producing TGF-β and stimulating collagen deposition.^37^

The elevated TGFβ expression in the ethanol+METH group from the IF analysis, along with increased fibrotic areas in the Picrosirius analysis (Figure 8(II)), also indicated that HSCs may be responsible for this proinflammatory response driven by M2 macrophages. In order to emulate the activation of HSCs in the context of M2-mediated inflammation, 3T3 cells were cultured and treated in DMEM without FBS, supplemented with the enriched medium derived from the supernatant of M2-polarized tumor-associated macrophages (TAMs). Surprisingly, NFkB gene expression was triggered not by M2 supernatant but by AuNPs + CARV treatments, which also decreased TGFβ gene expression, suggesting that fibroblastic cells can be activated in order to compensate rather than only to potentialize heavily M2-induced profibrotic microenvironments. CARV has been shown to enhance the expression of microRNA-200a and SMAD7, which inhibit TGF-β1 signaling, thereby reducing epithelial-mesenchymal transition (EMT) and collagen deposition in liver fibrosis models.^7,38^ Both SMAD-dependent and SMAD-independent pathways, including PI3K/AKT and MAPK pathways, are activated by TGFβ1.^39^ Additionally, CARV mitigates oxidative stress and HSC activation, further supporting its antifibrotic effects.^5,40^

In vivo, activated HSCs release chemokines that attract a diverse array of immune cells, including macrophages, neutrophils, natural killer (NK) cells, T cells, B cells, and innate lymphoid cells (ILCs). This recruitment establishes a self-sustaining cycle that continuously enhances and maintains the activated state of HSCs.^41,42^ Monocytes from peripheral blood are also attracted by HSCs’ chemokines, creating a whole new population of monocyte-derived macrophages in the liver known for its high CCR2 expression.^41^ Indeed, studies have demonstrated that in patients, the presence of M2 macrophages rises as liver fibrosis advances. This increase has been attributed to hepatic stellate cells (HSCs), which drive the process through the CCL2/CCR2 signaling pathway.^43^ Moreover, other studies suggest that monocyte-derived macrophages are even more activated than KCs in alcoholic injury and can be involved both in profibrogenic and antifibrogenic responses in different contexts.^44^ When human activated HSCs are cocultured with macrophages derived from peripheral blood mononuclear cells, they give rise to macrophages that produce IL-6 and TGF-β. However, inhibiting p38 in HSCs suppresses the development of these proinflammatory and profibrogenic macrophage phenotypes,^45^ proving that HSCs are capable of modulating macrophages in many different ways.

The effects of AuNPs + CARV treatment on HSCs were also noticeable by the expression of AKT and Pi3K in the immunofluorescence assay. In this assay, the treatment decreased both AKT and Pi3k expression. The Pi3K/AKT pathway is activated during HSCs’ proliferation and fibrotic state,^46^ as well as in hepatocyte proliferation.^47^ Similar findings were seen previously when only AuNPs were used to treat rats with alcoholic and methamphetamine liver injury,^1^ where AuNPs downregulated the activity of Kupffer cells and hepatic stellate cells, affecting the profile of their pro-inflammatory cytokines, oxidative stress, and fibrosis through modulation of AKT/PI3K and MAPK signaling pathways^1^ in order to mitigate liver injury caused by ethanol and methamphetamine. AuNPs effectively downregulated a critical pathway involved in HSC activation and collagen synthesis, resulting in a notable decrease in fibrotic markers, including procollagen I (PCI) and procollagen III (PCIII) mRNA levels. AuNPs also lowered TGF-β, a major contributor to HSC activation and fibrosis. By inhibiting the PI3K/AKT pathway, AuNPs reduced Kupffer cell activity, as indicated by decreased F4/80 mRNA expression. This suppression curtailed the release of pro-inflammatory cytokines (IL-1β, TNF-α), thereby further restricting HSC activation and fibrosis development. Additionally, AuNPs reduced oxidative stress markers like malondialdehyde and myeloperoxidase, suggesting that their ability to mitigate reactive oxygen species indirectly contributes to the inhibition of the PI3K/AKT pathway.

We believe this work encloses our research line on CARV and AuNPs applied to drug-induced inflammation, which started in 2016,^5^ as well as opens new perspectives on AuNP applications. These same nanoparticles were also used in another study aiming its diagnostic properties applied to immunofluorescence and flow cytometry, in which it was found that AuNPs can be used as reliable and inexpensive substitutes for Alexa Fluor 488.^48^ In this work, we took advantage of these properties for the uptake assay, which also suggests that AuNPs and CARV modulates macrophages not only through HSCs but acting directly on macrophage cells, decreasing the pro-inflammatory profile and blocking their profibrotic status.

Versatile technologies like these AuNPs, which serve both as therapeutic and diagnostic tools, can become the core of future research lines in nanomedicine applied to chronic inflammatory diseases and cancer. The integration of AuNPs with CARV for treating alcohol and methamphetamine-induced liver fibrosis exemplifies the convergence of nanomedicine and targeted liver disease therapies. AuNPs facilitate precise drug delivery to hepatic tissues, reducing systemic side effects while modulating macrophage polarization to promote antifibrotic M1 phenotypes. This strategy aligns with emerging nanomedicine approaches that combine therapeutic delivery with immune modulation to address fibrosis and inflammation. For instance, mannosylated nanoparticles delivering TGFβ1 siRNA have been shown to target profibrotic macrophages, effectively mitigating lung fibrosis by modulating immune responses.^49^ Additionally, the surface charge of nanoparticles influences their interaction with immune cells, affecting uptake and therapeutic outcomes.^50^ The antioxidant and anti-inflammatory synergy of CARV and AuNPs underscores the potential of multifunctional nanoparticles to address complex pathologies like liver fibrosis, which involve oxidative stress, inflammation, and fibrogenesis. These findings highlight the value of theranostic nanoparticles capable of both treatment and diagnostic monitoring, advancing precision medicine in liver disease and paving the way for innovative therapies targeting immune pathways and hepatic stellate cells.

## 4 Materials and Methods

### 4.1 Chemicals

Absolute ethanol (99.8% purity) was sourced from Vetec Quimica, Brazil. Methamphetamine (METH) was procured from Fisher Scientific in compliance with Brazilian law 10.357, enacted on December 27, 2001, which regulates the production, control, and inspection of chemical substances. Murine fibroblast cells (3T3, catalog No. CL-173) and macrophage cells (RAW 264.7; catalog No. TIB-71) were acquired from the American Type Culture Collection (ATCC, Manassas, VA). Dulbecco’s modified Eagle’s medium (DMEM) was purchased from Invitrogen Corporation (Carlsbad, CA), and fetal bovine serum (FBS) was obtained from Hyclone (Logan, UT). Recombinant mouse IFN-γ (catalog # 315-05) and IL-4 (catalog # 214-14) were supplied by PeproTech (Rocky Hill, NJ). Fluorescently labeled antibodies, including CD163-FITC (200 ng; Bioss, bs-2527R-FITC) and CD68-PerCP (200 ng; Abcam, ab220509), were used for specific staining. Additional antibodies targeting TGF-β, AKT, CD163, CD68, NFκB, IL-10, and PI3K were obtained from Santa Cruz Biotechnology Enterprise, Brazil. For nuclear staining, Fluoroshield Mounting Medium containing DAPI (20 mL) from ABCAM (Cambridge, U.K.) was utilized.

### 4.2 Cell Culture

The murine fibroblast cell line (3T3) and the RAW 264.7 mouse macrophage cell line were sourced from ATCC (Rockville, MD). The cells were cultured in an incubator at 37 °C with 5% CO_2_, using Dulbecco’s modified Eagle’s medium (DMEM, Gibco Laboratories, Grand Island, NY) enriched with 10% fetal bovine serum (FBS, Gibco Laboratories, Grand Island, NY) and 1% penicillin/streptomycin. Routine subculturing was performed every 2 weeks using trypsin/EDTA in phosphate-buffered saline (PBS).

### 4.3 AuNPs Production and Characterization

Spherical gold nanoparticles (AuNPs) with a size of 7.1 nm were synthesized and characterized following the methodology outlined by Gasparotto et al.^51^ Initially, all glassware was soaked overnight in a solution of KMnO_4_ and NaOH, followed by thorough rinsing with deionized water. The glassware was then dried and treated for 10 min with a mixture of H_2_O_2_ and H_2_SO_4_ (1:1 v/v). To prepare the AuNPs, gold chloride (6.80 mg) and poly(vinylpyrrolidone) (PVP, 0.20 g) were dissolved in 10 mL of water. Separately, glycerol (0.18 g) and NaOH (0.080 g) were dissolved in another 10 mL of water. The two solutions were combined, resulting in final concentrations of 1.0 mmol/L Au^3+^, 10 g/L PVP, 0.10 M NaOH, and 0.10 M glycerol. The mixture turned a deep crimson color, indicating the formation of AuNPs. The colloidal solution’s ultraviolet–visible absorption spectra were measured using an Evolution 60S UV–visible spectrophotometer (Thermo Scientific, MA), while fluorescence spectroscopy was performed using a Shimadzu RF-5301 PC spectrofluorophotometer (Kyoto, Japan).

### 4.4 In Vitro Assays

#### 4.4.1 Differentiation of RAW 264.7 Cells into the M2 Phenotype

The protocol for polarizing RAW 264.7 cells into M1 and M2 phenotypes was adapted from prior research.^52^ In brief, RAW 264.7 cells were seeded in a 6-well plate at a density of 0.2 × 10^6^ cells and cultured in DMEM for 24 h. To induce M2-like polarization, the cells were treated with 0.04 μg/mL of IL-4 in serum-free medium for 48 h. The resulting supernatant was collected and used as M2-conditioned medium (CM) for subsequent experiments. For M1-like polarization, RAW 264.7 cells were stimulated with 0.1 μg/mL lipopolysaccharide (LPS) and 0.1 μg/mL interferon-γ (IFN-γ) for 48 h. The polarized macrophages were characterized using flow cytometry and qPCR.

For analysis, M2-polarized RAW 264.7 cells were harvested, blocked with 0.5% BSA in PBS for 45 min, and then stained with anti-CD163-FITC (1:1000) or anti-CD68-PerCP (1:1000) antibodies. The labeled cells were analyzed using a FACSCalibur flow cytometer (BD Biosciences). The experiment was conducted in triplicate to ensure reproducibility and validate the findings.

#### 4.4.2 Viability Assay

In order to choose the most suitable doses of CARV and AuNPs for the upcoming assays, 3T3 cells were seeded in 12-well plates at a concentration of 0.1 × 10^6^ cells per well and cultured in DMEM for 24 h. A stock solution of CARV was prepared by diluting 2 mg of CARV into 10 mL of 1% DMSO. Then, cells were treated either with CARV (0.5, 1, 2, and 4 μg/mL) or AuNPs (0.5, 1, 2, and 4 μM/mL) during 24 and 48 h. After each period of treatment, equal volumes of cell aliquots and 0.5% (w/v) trypan blue solution were combined and allowed to incubate at room temperature for 5 min. Viable cell counts were determined using a hemocytometer.

#### 4.4.3 CARV and AuNPs In Vitro Uptake Assay

The uptake assay was carried out following another well-established protocol,^53^ with minor adaptations. In short, RAW 264.7 cells were plated on 12 mm glass coverslips at a density of 2 × 10^4^ cells per well in DMEM supplemented with 10% FBS. Following cell adhesion, treatments were applied for 24 and 48 h at 37 °C. The treatments included DMEM (control group), AuNPs (1 μM/mL), or a combination of AuNPs and CARV (1 μM/mL AuNPs + 1 μg/mL CARV). Since the AuNPs used in this study emit fluorescence, it was not necessary to conjugate CARV to another fluorophore.^48^ After washing, the cells were fixed using PBS, followed by 4% buffered paraformaldehyde (PFA; Sigma-Aldrich, Cat#158127). The coverslips were then carefully removed and placed onto glass slides using a DAPI-containing mounting medium (Abcam, Cat#ab104139). Fluorescence microscopy analysis was performed using an Axio Observer Z1 microscope (Zeiss).

#### 4.4.4 qPCR Assay for NFκB, TGFβ, IL-10, CD163, CD68, CD80, CD206, and ARG1 Gene Expression

Gene expression analysis was conducted on both 3T3 and RAW 264.7 cells to assess the M2 phenotype of polarized RAW 264.7 cells and to explore the potential impact of the conditioned medium from these M2 macrophages on the activation of 3T3 cells. For this, 3T3 cells were cultured in DMEM without FBS and mixed with enriched medium (supernatant from M2-polarized tumor-associated macrophages) in a 1:1 ratio, following a 48 h treatment with either AuNPs (1 μM/mL), CARV (1 μg/mL) or a combination of AuNPs and CARV (1 μM/mL AuNPs + 1 μg/mL CARV). Total RNA from both 3T3 and RAW 264.7 cells was extracted using Trizol reagent (Invitrogen, Cat#15596026) and purified with the SV Total RNA isolation system (Promega, Cat#Z3105), adhering to the manufacturer’s protocol. Next, cDNA was generated using a high-capacity RNA-to-cDNA kit (Applied Biosystems, Cat#4387406). Real-time amplification was carried out with PowerUp SYBR Green Master Mix (Applied Biosystems, Cat#A25742). The forward and reverse primer sequences (Thermo Fisher Scientific) utilized in this study are detailed in Table S1. All experiments were conducted in triplicate. Gene expression levels were normalized to the housekeeping gene β-actin and analyzed using the 2^–ΔΔCt^ method.

### 4.5 In Vivo Assays

#### 4.5.1 Animals

Thirty-six male Wistar rats, weighing between 250 and 280 g, were obtained from the animal facility of the Department of Biophysics and Pharmacology at the Federal University of Rio Grande do Norte (UFRN), Natal, Brazil. The rats were randomly divided into six groups of six animals each: (1) saline, (2) alcohol + saline, (3) alcohol + methamphetamine, (4) AuNPs + CARV 0.25 mg/kg, (5) AuNPs + CARV 0.5 mg/kg, and (6) AuNPs + CARV 1 mg/kg. The animals were housed in cages with free access to food and water, maintained under controlled temperature, humidity, and a 12-h light/dark cycle. All experimental procedures followed ethical guidelines for animal research and were approved by the UFRN Ethics Committee (approval number: 018/2015).

#### 4.5.2 Ethanol and METH-Induced Liver Injury Experimental Design

In order to induce liver damage by ethanol and METH, 30% ethanol solution (7 g/kg) was administered daily for 28 days to each rat by gavage as previously described.^5^ The healthy control group was exclusively treated with saline solution (0.9% NaCl). On the 22nd and 28th days of the study, a single oral dose of methamphetamine (METH, 10 mg/kg) was administered to the animals 3 h after ethanol treatment. In the nonmethamphetamine groups, saline solution was used as a substitute. Treatments were performed every day just 1 h before each ethanol gavage, following well-established protocols.^1,5^ AuNPs (724.96 mg/kg) were administered to the AuNP + CARV-treated groups, followed by CARV solution (0.25, 0.5, and 1 mg/kg) by gavage. The nontreated groups received only a saline solution.

On the 29th day, after a 12 h fasting period, euthanasia was carried out using intraperitoneal injections of ketamine (7.5 mL/kg, 50 mg/mL) and xylazine (2.5 mL/kg, 20 mg/mL). Once the animals were unconscious, a cardiac puncture was performed, and the liver was excised. Portions of the liver were frozen at −80 °C for subsequent cytokine and oxidative stress analysis. Additional liver fragments were preserved in 10% buffered formalin for histopathological examination.

#### 4.5.3 Transmission Electron Microscopy (TEM) for CARV and AuNP Uptake Assay by Hepatic Cells

To assess the in vivo uptake of AuNPs and CARV by hepatic cells, transmission electron microscopy (TEM) was conducted using a previously established protocol,^1^ in which tissue samples (0.5 cm) from each treatment group were fixed in Karnovsky’s solution (2.5% glutaraldehyde and 2.5% paraformaldehyde in 0.1 M cacodylate buffer) for about 4 h at 4 °C. Following fixation, the samples were rinsed four times with 0.1 M sodium cacodylate buffer (15 min per rinse). A solution of 1.6% potassium ferrocyanide (FCK) and 2% osmium tetroxide was then applied for 1 h in a darkroom. This was followed by two more rinses with 0.1 M sodium cacodylate buffer (15 min each) and two rinses with distilled water. The samples were stained en bloc with 0.5% uranyl acetate in a darkroom for 2 h under refrigeration. Dehydration was performed using a series of acetone concentrations, after which the samples were infiltrated and embedded in resin. Ultrathin sections (1 μm) were stained with toluidine blue and examined using a Zeiss EM 902 transmission electron microscope at 80 kV.

#### 4.5.4 Antioxidant Activity of AuNPs and CARV

The antioxidant properties of AuNPs and CARV were assessed by measuring reduced glutathione (GSH) levels, malondialdehyde (MDA) production, and myeloperoxidase (MPO) activity inhibition. Liver tissue samples were collected as previously outlined and stored at −80 °C until analysis. Following homogenization and centrifugation (2000*g* for 20 min), MPO activity was quantified using a well-established colorimetric technique.^5^ To measure the rise in free radicals within the liver samples, malondialdehyde (MDA) levels were determined using the method outlined by Esterbauer and Kevin in 1990.^54^ Liver tissue samples were mixed with Tris-HCl buffer in a 1:5 (w/v) ratio and finely chopped with scissors for 15 s on an ice-cooled surface. The mixture was then homogenized for 2 min using an automated potter homogenizer and centrifuged at 2500*g* for 10 min at 4 °C. The resulting supernatants were analyzed to quantify malondialdehyde (MDA) levels, with results reported as nanomoles of MDA per gram of tissue. Reduced glutathione (GSH) content was measured following the protocol established by de Araújo et al. in 2017.^20^ Liver samples (six per group) were stored at −80 °C until analysis. Tissue homogenates were prepared by mixing 0.25 mL of a 5% liver solution (in 0.02 M EDTA) with 320 mL of distilled water and 80 mL of 50% trichloroacetic acid (TCA). The mixture was centrifuged at 3000 rpm for 15 min at 4 °C. From the supernatant, 400 mL was combined with 800 mL of 0.4 M Tris buffer (pH 8.9) and 20 μL of 0.01 M DTNB. Absorbance readings were taken at 420 nm, and GSH levels were calculated and expressed as micrograms of GSH per milligram of tissue.

#### 4.5.5 Cytokine Analysis

Liver samples (three per group) were stored at −80 °C until analysis. The tissue was homogenized and prepared following the method outlined by Safieh-Garabedian et al.^55^ Concentrations of IL-1β (detection range: 62.5–4000 pg/mL; sensitivity: 12.5 ng/mL), IL-10 (detection range: 62.5–4000 pg/mL; sensitivity: 12.5 ng/mL), and TNF-α (detection range: 62.5–4000 pg/mL; sensitivity: 50 ng/mL) in the liver samples were measured using a commercial ELISA kit (R&D Systems, Minneapolis, MN), as previously described. Absorbance readings for all samples were taken at 490 nm using UV–vis spectrophotometry.

#### 4.5.6 Histopathological Analysis

Liver samples intended for histological examination were preserved in 10% buffered formalin, dehydrated, and embedded in paraffin using standard procedures.^1,5,53^ Three sections (5 μm thick) from each sample (six animals per group) were prepared for hematoxylin-eosin (H&E) staining and analyzed under a light microscope (40× magnification, Nikon E200 LED). Steatohepatitis was evaluated in a double-blind manner by pathologists, following established criteria,^5,56^ which included assessing fatty degeneration (fat accumulation in liver cells), inflammation, necrosis, and fibrosis, as detailed in Table S2. The average scores were used for statistical comparisons.

To quantify collagen content (fibrosis), liver samples were stained using a Picrosirius Red staining kit (1% Sirius red in saturated picric acid; EasyPath, Indaiatuba, Brazil). Light microscope images (200×) of large centrilobular veins and portal tracts (≥150 μm) were analyzed. Approximately 20 polarized light microscopy images per specimen were captured using an Olympus BX60 microscope (200×) and processed with a color threshold detection tool in ImageJ (National Institutes of Health). Positive and negative controls were included in each sample batch. Results are expressed as the percentage of positively stained areas. Contrast index measurements were calculated as (selected area × 100)/total area, with three samples analyzed per animal.

To assess alcohol-induced liver injury, blood samples were collected via cardiac puncture, centrifuged at 3000*g* for 10 min, and the resulting supernatants were used to measure alanine aminotransferase (ALT), aspartate aminotransferase (AST), albumin, and γ-glutamyl transferase (GGT) levels using an automated analyzer (FDC4000; Fuji Medical Systems, Tokyo, Japan). Data are presented as means ± standard error of the mean (SEM).

#### 4.5.7 Immunofluorescence Microscopy for AKT, TGFβ, CD163, CD86, NFκB, IL-10, and PI3K

For immunofluorescence assay, only samples from saline, alcohol+meth, and AuNP+CARV 1 mg/kg groups were used. Three tissue sections from each animal (six animals per group) were deparaffinized using xylene and rinsed through a graded ethanol series followed by PBS. Antigen retrieval was achieved by heating the sections in 10 mM sodium citrate buffer containing 0.05% Tween 20 at 95 °C for 40 min. To minimize autofluorescence, the sections were treated with 0.1% Sudan black in 70% alcohol for 40 min at room temperature (RT). The sections were then incubated overnight at 4 °C in a humidified chamber with primary antibodies against AKT, TGFβ, CD163, CD86, NFκB, IL-10, and PI3K (Santa Cruz Biotechnology), diluted 1:400 in Diamond antibody diluent (Cell Marque, CA). After three washes with PBS containing 0.2% Triton X-100 (5 min each), the sections were incubated with Alexa Fluor 488-conjugated goat antirabbit secondary antibody (1:400 in Diamond antibody diluent) for 1 h at 22 °C in a humidified chamber. Finally, the sections were coverslipped using DAPI-containing mounting medium (ab104139, Abcam). Fluorescent images were captured using Zeiss Zen Lite software (Carl Zeiss, Germany), and the arithmetic mean fluorescence intensity of at least 10 images per section (200× and 400× magnification) was used for statistical analysis.

### 4.6 Statistical Analysis

All in vitro experiments were reported as the mean ± standard deviation (SD) from five independent assays, each with a minimum of three replicates. The in vitro analyses were conducted in a randomized and double-blind manner by two researchers (R.A. & V.G.). Similarly, ex vivo data (histopathology and immunofluorescence) were evaluated in a double-blind fashion by the same authors. Experimental designs ensured randomized grouping with equal sample sizes. Groups were formed using independent values exhibiting less than 20% variance. Statistical analysis was performed only for experiments with group sizes of *n* ≥ 5 independent values. All statistical evaluations were carried out using Prism 6.01 software (GraphPad Software, RRID:SCR_002798). The normality of data distribution was assessed using the Shapiro-Wilk test, while the Levene test was employed to confirm variance homogeneity. For multiple group comparisons, one-way ANOVA was applied, followed by post hoc Dunnett tests. Non-normally distributed data were analyzed using the Kruskal–Wallis test with Dunn’s correction. A *p*-value of less than 0.05 was considered statistically significant.

## 5 Conclusions

In this study, AuNPs and CARV proved to ameliorate profibrotic inflammatory processes by acting on HSCs and modulating macrophage polarization toward less fibrogenic M1 phenotype.

## Data Availability

The data underlying this study are available in the published article and its Supporting Information.

## Supporting Information Available

Figure S1: Fluorescence intensity from each treated group of the uptake assay; Table S1: Details of the mouse primer sequences, amplicon sizes, and annealing temperatures employed in this study; and Table S2: Histopathological scores (PDF)

## Notes

The animal study protocol received approval from the Ethics Committee on the Use of Animals (CEUA) at the Federal University of Rio Grande do Norte (UFRN, Brazil) (Approval Number: 018/2015).

## Abbreviations

ALD: alcoholic liver disease
MILD: methamphetamine-induced liver disease
METH: methamphetamine
CARV: carvedilol
AuNPs: gold nanoparticles
HSCs: hepatic stellate cells
GSH: glutathione
MDA: malondialdehyde
MPO: myeloperoxidase
TGF-β: transforming growth factor β
TNF-α: tumor necrosis factor α
IL-1β: interleukin-1β
IL-10: interleukin 10
NFκB: nuclear factor kappa B
AKT: protein kinase B
PI3K: phosphoinositide 3-kinase
LPS: lipopolysaccharide
DMEM: Dulbecco’s modified Eagle’s medium
PBS: phosphate-buffered saline
FBS: fetal bovine serum
ELISA: enzyme-linked immunosorbent assay
RT: room temperature
TEM: transmission electron microscopy
CCR2: CC chemokine receptor 2
CCL2: CC chemokine ligand 2
CCR5: CC chemokine receptor 5
TLC: thin-layer chromatography
H&E: hematoxylin and eosin
GGT: γ-glutamyl transferase
ALT: alanine aminotransferase
AST: aspartate aminotransferase
ARG1: arginase-1
DAPI: 4′,6-diamidino-2-phenylindole
