# Supplementary material for: Gold Nanoparticles (AuNPs) Coadministered with a β-Blocker Prevent Liver Fibrosis Caused by Ethanol and Methamphetamine in Rats by Downregulating the Expression of M2 Macrophages
Source: ACS Omega. 2025 Apr 8;10(15):14924–39. doi: 10.1021/acsomega.4c10118 (PMC12019731; doi:10.1021/acsomega.4c10118)
Supplement: Supplementary file 1 — ao4c10118_si_001.pdf [file ao4c10118_si_001.pdf]

Supporting Information files

Paper title: Gold Nanoparticles (AuNPs) co-administered with a β-blocker prevent liver fibrosis caused by ethanol and methamphetamine in rats by down-regulating the expression of M2 macrophages.

Authors: Vinícius B. Garcia[1], Luiz H. S. Gasparotto[2], Aurigena A. de Araujo[3], Renata F. C. Leitão[4], Gerly A. C. Brito[4], Natalia Feitosa Vilar [1], Emily Lima Oliveira[1], Paulo M. M. Guedes[5], Raimundo F. de Araújo Júnior[1] *

1. Inflammation and Cancer Research Laboratory, Department of Morphology, Federal University of Rio Grande do Norte (UFRN), Natal, RN, Brazil

2. Institute of Chemistry, Federal University of Mato Grosso (UFMT), Cuiaba, MT, Brazil

3. Department of Pharmacology, Federal University of Rio Grande do Norte (UFRN), Natal, RN, Brazil

4. Department of Morphology, Postgraduate Program in Morphology, Federal University of Ceará (UFC), Fortaleza, CE, Brazil

5. Department of Microbiology and Parasitology, Federal University of Rio Grande do Norte (UFRN), Natal, RN, Brazil

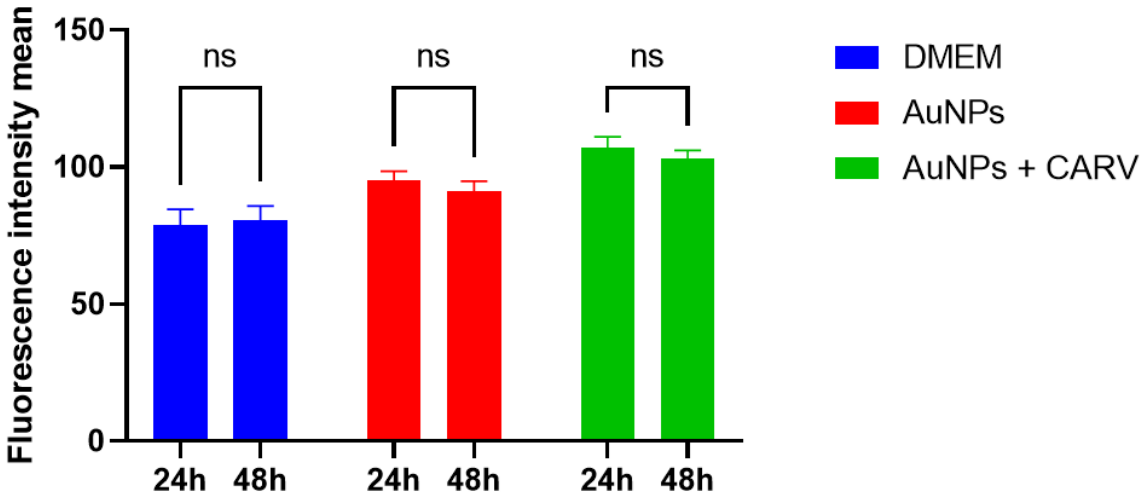

Figure S1. Fluorescence intensity means comparison between the 24-hour and 48-hour uptake assays. No difference was considered statistically significant (ns). All data are reported as mean ± standard deviation (SD) from four independent experiments, each conducted with at least two replicates. Data were analyzed using two-way ANOVA followed by post hoc Dunnett's (a) or Sidak's (b) correction.

Table S1: Mouse primers sequence, amplicon size and annealing temperature used in this study.

Target	5' --> 3' (Forward)	3' --> 5' (Reverse)	Product size (pb)	Annealing temperature (ºC)	Melting temperature (ºC)
NFkB	CGAGCGAGCTCTAGT TCCTG	AGCTCTGATCCCC CTAAGCA	527	52	60
TGFb	CCGCAACAACGCCAT CTATG	GGATCCACTTCCA ACCCAGG	590	52	60
IL-10	GGGTGAGAAGCTGAA GACCC	AGGACACCATAGC AAAGGGC	597	52	60
CD163	TTGGATGTGGATCTG CGCTT	CGCCTGCCAGACG AATATCT	522	52	60
CD68	AAAGGCCGTTACTCT CCTGC	ACTCGGGCTCTGA TGTAGGT	544	52	60
CD80	ACAACAGCCTTACCT TCGGG	CTGGAAGTCTGGC ACTCAGG	504	52	60
CD206	GAGCCTGGAAAGAGC TGTGT	ACCCTCCGGTACT ACAGCAT	567	52	60
ARG-1	CAATCGATGCACCTG CCATG	CGGCTGTGCATCA TACAACG	576	52	60

Table S2: Histopathological scores used in this study

Histological findings	Score system	Meaning
Liver Steatosis	1	<25% of ballooned hepatocytes
	2	25-50% of ballooned hepatocytes
	3	50-75% of ballooned hepatocytes
	4	>75% of ballooned hepatocytes
Inflammatory infiltration	1	Absent
	2	Mild infiltration
	3	Moderate infintration
	4	Intense infiltration
Necrotic sites	1	No necrotic sites
	2	One or more necrotic sites
